# Frequency and Risk Factors for Under- and Over-Treatment in Stroke Prevention for Patients with Non-Valvular Atrial Fibrillation in General Practice

**DOI:** 10.1371/journal.pone.0067806

**Published:** 2013-07-05

**Authors:** Derk L. Arts, Stefan Visscher, Wim Opstelten, Joke C. Korevaar, Ameen Abu-Hanna, Henk C. P. M. van Weert

**Affiliations:** 1 Department of General Practice, Academic Medical Centre, Amsterdam, The Netherlands; 2 Department of Medical Informatics, Academic Medical Centre, Amsterdam, The Netherlands; 3 Netherlands Institute for Health Services Research, Utrecht, The Netherlands; 4 Dutch College of General Practitioners, Utrecht, The Netherlands; Maastricht University Medical Center, Netherlands

## Abstract

**Objective:**

To determine adequacy of antithrombotic treatment in patients with non-valvular atrial fibrillation. To determine risk factors for under- and over-treatment.

**Design:**

Retrospective, cross-sectional study of electronic health records from 36 general practitioners in 2008.

**Setting:**

General practice in the Netherlands.

**Subjects:**

Primary care physicians (n = 36) and patients (n = 981) aged 65 years and over.

**Main Outcome Measures:**

Rates of adequate, under and over-treatment**,** risk factors for under and over-treatment.

**Results:**

Of the 981 included patients with a mean of age 78, 18% received no antithrombotic treatment (under-treatment), 13% received antiplatelet drugs and 69% received oral anticoagulation (OAC). Further, 43% of the included patients were treated adequately, 26% were under-treated, and 31% were over-treated. Patients with a previous ischaemic stroke were at high risk for under-treatment (OR 2.4, CI 1.6–3.5), whereas those with contraindications for OAC were at high risk for over-treatment (OR 37.0, CI 18.1–79.9). Age over 75 (OR 0.2, CI: 0.1–0.3]), diabetes (OR 0.1, CI: 0.1–0.3), heart failure (OR 0.2, CI: 0.1–0.3), hypertension (OR 0.1, CI: 0.1–0.2) and previous ischaemic stroke (OR 0.04, CI: 0.02–0.11) protected against over-treatment.

**Conclusions:**

In general practice, CHADS_2_-criteria are being used, but the antithrombotic treatment of patients with atrial fibrillation frequently deviates from guidelines on this topic. Patients with previous stroke are at high risk of not being prescribed OAC. Contraindications for OAC, however, seem to be frequently overlooked.

## Introduction

Every patient over the age of 65 with atrial fibrillation (AF) existing over 48 hours, needs anti-thrombotic treatment, as AF increases the risk of stroke fivefold in untreated patients [1]. Moreover, stroke caused by AF is also associated with increased severity [2], thus leading to increased mortality and disability. Stroke risk reduction is currently achieved by administering antithrombotic therapy to patients with AF. Antithrombotic therapy with oral anticoagulation (OAC) decreases the risk of stroke by approximately 60% and by approximately 20% with antiplatelet drugs in patients with AF [3], [4].

Because OAC increases the risk of major bleeding, current national and international guidelines on AF incorporate stroke risk stratification schemes to calculate stroke risk, balance benefits and risks of OAC-treatment, and recommend appropriate therapy [5], [6]. The CHADS_2_ score is a commonly used stroke risk stratification scheme, which is calculated by assigning 1 point each for the presence of congestive heart failure, hypertension, age 75 years or older, and diabetes mellitus; and by assigning 2 points each for history of stroke or transient ischaemic attack (TIA) [7], [8]. Thromboprophylaxis with OAC is recommended in patients with moderate to high stroke risk (CHADS_2_ score ≥2), while antiplatelet therapy is recommended for patients with low risk [3], [5], [7].

Former hospital based studies showed that OAC prescription rates for high-risk patients vary from 52% to 67% [9], [10]. A study among Dutch general practitioners (GPs), internists, and cardiologists confirmed these findings of secondary care studies, albeit with somewhat higher compliance rates (67–72%) [11]. Because general practitioners in the Dutch healthcare system provide longitudinal and integral care to their patients, they are in a good position to provide a patient-tailored treatment.

We report on adequacy of antithrombotic treatment for patients over 65 years old, using routinely collected data from the GP’s electronic healthcare records (EHR). Furthermore, we aim to identify risk factors for inadequate treatment.

## Methods

### Data Collection

Data were extracted from EHR of general practices that participated in the Netherlands Information Network of General Practice (LINH) in 2008 [12]. The LINH forms a geographically well-distributed network of GPs in The Netherlands. The LINH patient population has a stable size and its age distribution and gender ratios are similar to those of the general Dutch population. The database holds International Classification of Primary Care (ICPC) coded longitudinal data on morbidity, prescribing, and referrals of about 340,000 individuals. For this study, practices were only eligible for extraction when their data met the necessary quality requirements, which pertained to the number of records present per individual patient, diagnoses present per individual patient, and number of registered ICPC codes (60% of all patient contacts). The number of ICPC codes per patient was compared to similar practices and counts of previous years to ensure completeness of the registration. These data had to be available for a period of at least 3 years.

### Patients

Characteristics were extracted for all patients aged 65 years and older who suffered from AF at the end of 2008. Medication prescriptions, including OAC and antiplatelet drugs, relevant comorbidities and contraindications for OAC were selected from the database. Patients with valvular abnormalities were excluded. Of all patients with atrial fibrillation, those without any antithrombotic medication were identified using R [13]. For these patients, correctness of the diagnosis of atrial fibrillation was checked with the treating GP and patients with wrongly recorded atrial fibrillation were excluded.

### Ethics Statement

In the Netherlands, there is no need to obtain consent when only registry data obtained from routine care and without patient identifying information are used, as is stated in the selection criteria for the Medical Research Involving Human Subjects Act (WMO) [14].

### Treatment Evaluation

In order to evaluate treatment adequacy and under and over-treatment, we first determined the recommended treatment for every patient according to the CHADS_2_ stroke risk stratification scheme [8], [15]. If contraindications for OAC were present, the recommended treatment was antiplatelet drugs. If no contraindications were present, the CHADS_2_ score was calculated using ICPC-coded diagnoses. The recommended treatment was antiplatelet drugs for scores <2, and OAC for scores ≥2. We then compared the recommended treatment with the actual treatment. We classified adequacy of treatment as follows: patients who received no antithrombotic treatment and patients with CHADS_2_ scores ≥2 who received only antiplatelet drugs were classified as under-treated. Patients with contraindications for OAC and/or with CHADS_2_ scores <2 who were treated with OAC were included in the over-treatment group. The remaining patients were classified as adequately treated.

### Statistical Analysis

Variables were initially selected based on the HAS-BLED score [16] and our clinical judgement of their relevancy to adequacy of treatment: conditions incorporated in the CHADS_2_-score, contraindications for OAC and conditions that might be responsible for withholding indicated treatment or other conditions than atrial fibrillation that warranted OAC treatment. These variables were: age, gender, diabetes, hypertension, congestive heart failure, ischaemic heart disease, previous stroke, epilepsy, Parkinson’s disease, cognitive impairment, risk of falling (history of falling or use of sedatives), established contraindications for OAC (haemorrhagic stroke, history of large bleeds, kidney or liver failure (including alcoholism), coagulation diseases), deep venous thrombosis or pulmonary embolism and the use of heparins. We were not able to accurately assess labile INR’s and actual hypertension >160 mm Hg. Parkinson’s disease, epilepsy and risk of falling were clustered into one variable, as were contraindications for OAC. We compared these variables within the three treatment outcome groups and calculated chi-squares to test for association.

To identify independent associations with adequate treatment we applied multivariate logistic regression in which each treatment outcome group was compared against the remaining groups. We used backward stepwise variable elimination based on the Akaike Information Criterion, and checked for presence of collinearity using the variance inflation factor [17]. The model’s accuracy was measured using the standard bootstrap procedure with 100 bootstrap samples and the percentile bootstrap confidence intervals. All analyses were performed with the R language [13] using the RMS library for R [18]. The Area under the curve (AUC) was determined to assess the model’s discriminating power. The AUC ranges from 0.5 (no discrimination) to a theoretical maximum of 1. Perfect discrimination corresponds to an AUC of 1 and is achieved if the scores for all the cases are higher than those for all the non-cases, with no overlap.

## Results

### Patient Characteristics

**Figure 1:**
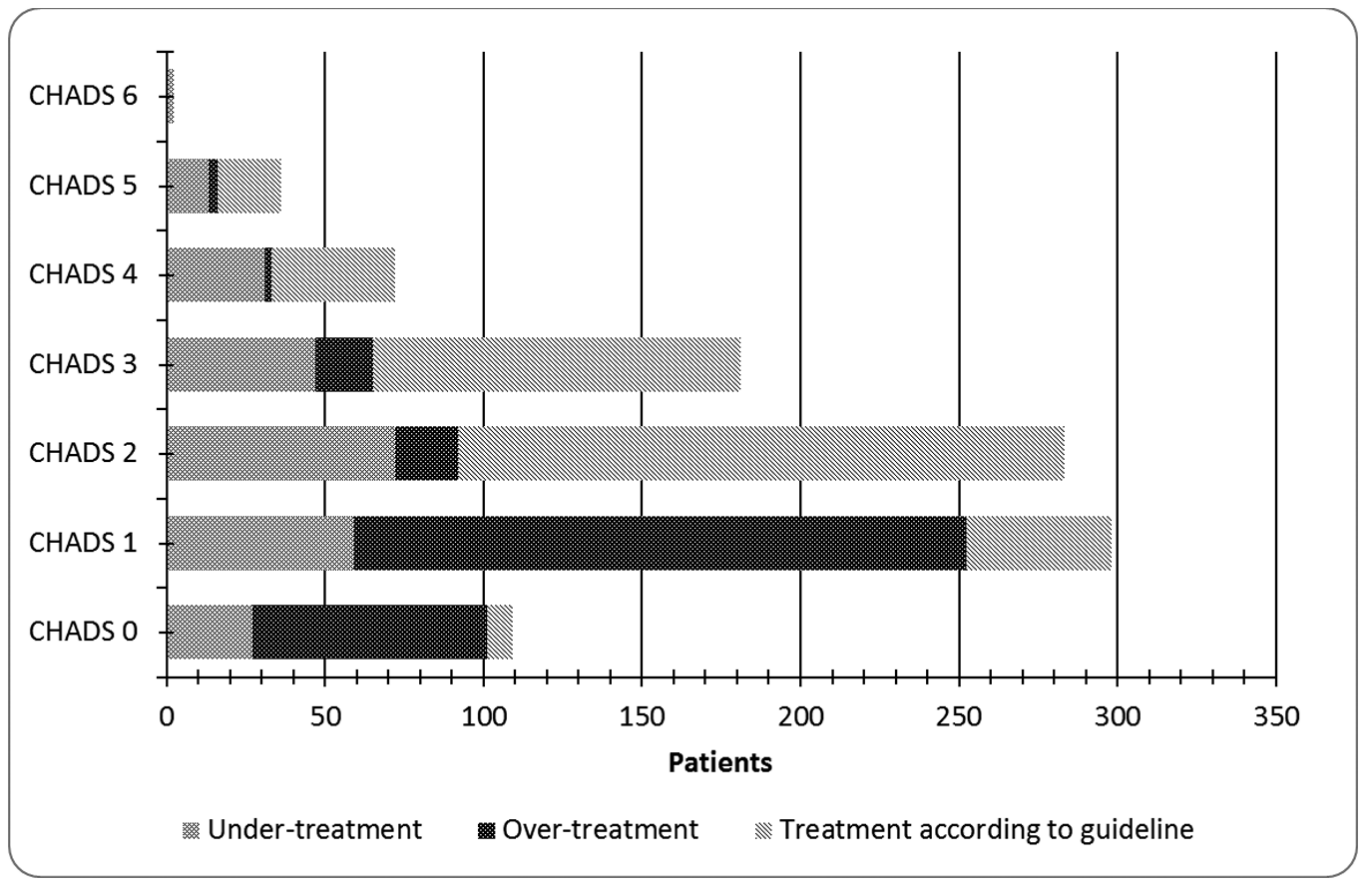
Number of patients and proportions of under- and over-treatment per CHADS_2_ score in patients with atrial fibrillation.

After selecting practices with the required availability of three years of complete data, 36 general practices with 148,528 patients remained. This resulted in a total number of 981 patients with AF. Mean age was 78 years and 46% were men. CHADS_2_ score was 1.9 on average, with only 2 patients scoring 6 points. The majority (59%) had a CHADS_2_ score ≥2 and 69% were treated with OAC (Figure 1). More than 16% of all included patients had one or more contraindications for OAC use. One or more comorbidities were present in 81% of patients, with hypertension being the most prevalent (57%) (Table 1).

**Table 1:** Population and proportions per treatment outcome.

	n	Perc.	n	Perc.	n	Perc.		n	Perc.		
	All		Under-treatment		Over-treatment			Adequate treatment*			Significance
**Overall**	981		251	25.59%	310		31.60%	420		42.81%	
**Age 65 - 75**	383	39.04%	88	35.06%	181		58.39%	114		27.14%	<0.001
**Age >75**	598	60.96%	163	64.94%	129		41.61%	306		72.86%	<0.001
**Men**	448	45.67%	110	43.82%	171		55.16%	167		39.76%	<0.001
**Co-morbidity present**	792	80.73%	211	84.06%	175		56.45%	405		96.43%	<0.001
Diabetes	217	22.12%	52	20.72%	23		7.42%	142		33.81%	<0.001
Heart failure	303	30.89%	85	33.86%	33		10.65%	158		37.62%	<0.001
Ischaemic heart disease	184	18.76%	40	15.94%	52		16.77%	92		21.90%	0.09
Hypertension	756	77.06%	145	57.77%	105		33.87%	306		72.86%	<0.001
Ischaemic stroke	120	12.23%	50	19.92%	10		3.23%	60		14.29%	<0.001
Falling, epilepsy, Parkinson’s	150	15.29%	42	16.73%	45		14.52%	63		15.00%	0.75
Sedative use	228	23.24%	53	21.12%	74		23.87%	101		24.05%	0.65
**Contraindications for OAC** * **,** **	82	8.36%	26	10.36%	52		16.77%	4		0.95%	<0.001
Haemorrhagic stroke	2	0.20%	1	0.40%	1		0.32%	0		0.00%	0.13
Large bleeding	32	3.26%	5	1.99%	27		8.71%	0		0.00%	<0.001
Liver failure	3	0.31%	1	0.40%	1		0.32%	1		0.24%	0.93
Kidney failure	1	0.10%	1	0.40%	0		0.00%	0		0.00%	0.23
Coagulation disease	5	0.51%	1	0.40%	3		0.97%	1		0.24%	0.38
Cognitive impairment	41	4.18%	17	6.77%	22		7.10%	2		0.48%	<0.001
**CHADS_2_<2**	407	41.49%	86	34.26%	267		86.13%	54		12.86%	<0.001
**CHADS_2_≥2**	574	58.51%	165	65.74%	43		13.87%	366		87.14%	<0.001

* According to the Dutch GP guideline.

** Values displayed in this row reflect one or more contraindications.

### Treatment

A total of 420 patients (43%) were treated adequately; 251 patients (26%) were under-treated, 172 out of these 251 (69%) patients did not receive any antithrombotic treatment and 79 (31%) patients had a CHADS_2_ scores ≥2 but were on antiplatelet drugs. A total of 310 patients (32%) were over-treated, among whom 267 (86%) patients had CHADS_2_ scores <2, but were on OAC (table 1). Of all patients treated with OAC, almost 8% had contraindications.

### Risk Factors

Table 2 shows factors associated with treatment adequacy. No evidence for collinearity between variables was discovered. Presence of contraindications for OAC was the only risk factor that significantly increased the chance of over-treatment. Independent protective factors against over-treatment were age >75, presence of diabetes, heart failure, hypertension, and previous ischaemic stroke or TIA (all CHADS_2_ criteria). Previous stroke or TIA increased the risk for under-treatment, as did heart failure to a lesser degree. Factors that significantly decreased the chance of under-treatment were not found.

**Table 2:** Models for over- and under-treatment.

1. Model for over-treatment					
Variable	OR	95% CI			AUC††
**Age >75**	0.19†	0.13	–	0.28	0.89
**Diabetes**	0.14†	0.08	–	0.25	
**Heart failure**	0.15†	0.09	–	0.25	
**Hypertension**	0.14†	0.10	–	0.20	
**Previous stroke/TIA**	0.04†	0.02	–	0.11	
**Contraindications for OAC** *	36.98†	18.11	–	79.93	
**Removed stepwise** **					
**Female sex**	0.71	0.50	–	1.02	
**Ischaemic heart disease**	0.84	0.53	–	1.33	
**Neurological disease** ***	1.09	0.64	–	1.84	
**Sedative use**	1.20	0.78	–	1.83	
**2. Model for under-treatment**					
**Variable**	**OR**	**95% CI**			**AUC** ††
**Previous stroke/TIA**	2.35†	1.57	–	3.48	0.54
**Removed stepwise** **					
**Age >75**	1.10	0.81	–	1.51	
**Female sex**	1.11	0.82	–	1.50	
**Diabetes**	0.91	0.62	–	1.31	
**Heart failure**	1.45	1.03	–	2.03	
**Ischaemic heart disease**	0.76	0.50	–	1.14	
**Hypertension**	1.19	0.86	–	1.65	
**Neurological disease** ***	1.07	0.70	–	1.60	
**Contraindications for OAC** *	1.22	0.72	–	2.03	
**Sedative use**	0.76	0.52	–	1.09	

* Haemorrhagic stroke, large bleed in history, liver failure, kidney failure, coagulation disease, cognitive impairment (dementia, psychosis).

** As selected by AIC. Odds ratios are reported for the complete model.

*** Parkinson’s disease, epilepsy, history of falling.

† P<0.01.

†† Area under the curve or concordance-index: a measure of discriminating power of the model.

## Discussion

In this analysis of GPs’ electronic healthcare records, we have shown antithrombotic treatment in patients with non-valvular AF to be inadequate. Over half of patients did not receive the recommended treatment. All CHADS_2_ criteria (age >75, diabetes, heart failure, hypertension, previous stroke) independently reduced the risk of over-treatment, as was to be expected. Contraindications for OAC however increased this risk. Previous stroke or TIA increased the chance for under-treatment and reduced the change of over treatment. Female sex is associated with lower OAC rates in other studies [15], [18], but our data do not support these findings.

Although our study was performed in primary care, demographic characteristics of our study population were comparable with other studies on this topic [3], [19], [20]. Mean age was slightly higher, which can be attributed to the fact that we only included patients of 65 years or older. Comorbidity rates were comparable, with the exception of ischaemic heart disease, which showed markedly lower prevalence (19% vs. 28%) [11], [20], [21]. This can be explained by the fact that other studies were mainly performed in secondary care.

We found antithrombotic treatment adequacy was low, but comparable to other studies on antithrombotic treatment in patients with AF [3], [11], [19], [20]. These studies compared stroke risk scores to antithrombotic prescriptions and did not take contraindications in individual patients into account. This led to patients being classified as adequately treated when they were actually over-treated due to contraindications for OAC, thus leading to higher rates of adequate treatment.

Our data suggest that contraindications for OAC are not always evaluated, which we ascribe to the fact that contraindications can be easily overlooked in busy everyday practice. We found no significant independent associations between treatment adequacy and sedative use, history of epilepsy/falling, or ischaemic heart disease. Our results suggest previous stroke/TIA increases the risk of under-treatment, but this could be due to problems in the registration of contraindications (mainly stroke and renal failure). It is possible that some patients actually suffered from a haemorrhagic stroke, in which case the treatment would have been adequate.

### Strengths and Limitations

We studied antithrombotic treatment of atrial fibrillation in real life, by using routinely registered data. These data were subjected to several rigorous quality checks, but our evaluation was slightly hampered, by a lack of detail and incompleteness of diagnostic coding for two diagnoses: Diagnoses of cerebral haemorrhage and ischaemic stroke are both classified as sub-codes of the ICPC code K90, but in the majority of cases, no sub-codes were specified. Given the much greater prevalence of ischaemic stroke, we classified all unknown cases of stroke as ischaemic. Also, renal failure appeared to be underreported, as we only found 2 cases of renal failure, which is lower than expected in this population. This probably caused some misclassification that might have led to inflation of reported under-treatment. On the other hand, the risk of being over-treated due to the presence of contraindications for OAC is probably larger than reported. Hypertension is both an indication, as well as a contra-indication for OAC, as it is incorporated in both the CHADS_2_ and the HASBLED score. Because we were unable to assess the exact blood pressure, we considered treated hypertension as an indication. Instable INR’s are not coded in the GP’s EHR as treatment with OAC is monitored by a separate service in the Netherlands. This might have led to slight over reporting of overtreatment.

### Implications for Clinical Practice and Research

By including not only comorbidities and medication, but also contraindications for individual patients, we have come close to a real-life treatment scenario. GPs should be more aware of contraindications for OAC, as stroke risk does not justify the added risk of major bleeding. They should also be aware of the presence of a previous ischaemic stroke, as this is always a reason to prescribe OAC. Lastly, we would like to stress the importance of accurate diagnostic coding and electronic record keeping. This will allow for more accurate research and will enable the use of decision support systems to support caretakers in applying complex guidelines. Decision support systems might play a vital role in the improvement of stroke prevention, as has been stated and validated in other areas of medicine [21], [22]. Further research should be done to assess the use of these systems in medical practice, as clues from the medical history seem to be easily overlooked.

### Conclusions

In Dutch general practice, the antithrombotic treatment of patients with atrial fibrillation frequently deviates from guidelines on this topic. Patients with previous stroke are at high risk of not being prescribed OAC. Contraindications for OAC, however, seem to be frequently overlooked, resulting in over-treatment, which shows tailoring treatment remains important.
